# Corticomuscular coherence during upright standing in unilateral transfemoral amputees

**DOI:** 10.1093/braincomms/fcaf238

**Published:** 2025-06-14

**Authors:** Britta Meyer, Thomas Krauskopf, Katharina Fuchs, Marvin Beusterien, Lukas Klein, Marc Mueller, Tonio Ball, Georg W Herget, Natalie Mrachacz-Kersting, Vinzenz von Tscharner, Carsten Mehring, Thomas Stieglitz, Cristian Pasluosta

**Affiliations:** Laboratory for Biomedical Microtechnology, Department of Microsystems Engineering, University of Freiburg, Freiburg 79110, Germany; Institute for Sport and Sport Science, University of Freiburg, Freiburg 79117, Germany; Laboratory for Biomedical Microtechnology, Department of Microsystems Engineering, University of Freiburg, Freiburg 79110, Germany; BrainLinks-BrainTools Center, University of Freiburg, Freiburg 79110, Germany; Laboratory for Biomedical Microtechnology, Department of Microsystems Engineering, University of Freiburg, Freiburg 79110, Germany; Laboratory for Biomedical Microtechnology, Department of Microsystems Engineering, University of Freiburg, Freiburg 79110, Germany; Department of Orthopaedics and Trauma Surgery, University Medical Center Freiburg, Freiburg 79106, Germany; Sanitätshaus Pfänder, Freiburg 79111, Germany; BrainLinks-BrainTools Center, University of Freiburg, Freiburg 79110, Germany; Neuromedical AI Lab, Department of Neurosurgery, Medical Center—University of Freiburg, Faculty of Medicine, Freiburg 79106, Germany; Department of Orthopaedics and Trauma Surgery, University Medical Center Freiburg, Freiburg 79106, Germany; Institute for Sport and Sport Science, University of Freiburg, Freiburg 79117, Germany; BrainLinks-BrainTools Center, University of Freiburg, Freiburg 79110, Germany; Human Performance Laboratory, University of Calgary, Calgary, Canada T2N 1N4; BrainLinks-BrainTools Center, University of Freiburg, Freiburg 79110, Germany; Faculty of Biology, University of Freiburg, Freiburg 79104, Germany; Bernstein Center Freiburg, University of Freiburg, Freiburg 79104, Germany; Laboratory for Biomedical Microtechnology, Department of Microsystems Engineering, University of Freiburg, Freiburg 79110, Germany; BrainLinks-BrainTools Center, University of Freiburg, Freiburg 79110, Germany; Bernstein Center Freiburg, University of Freiburg, Freiburg 79104, Germany; Laboratory for Biomedical Microtechnology, Department of Microsystems Engineering, University of Freiburg, Freiburg 79110, Germany; BrainLinks-BrainTools Center, University of Freiburg, Freiburg 79110, Germany

**Keywords:** postural control, amputee, corticomuscular coherence, sensorimotor

## Abstract

Patients with a lower limb amputation suffer from an impaired balance control and thereby are at a higher risk to fall. To cope with this deficit, they adapt their neuromuscular system by modifying biomechanical and neuromuscular structures. In this study, we investigated changes in corticomuscular coherence between the motor cortex and muscles of the trunk and the intact lower leg. We recorded electroencephalogram (EEG) and electromyogram (EMG) data from 10 unilateral transfemoral amputees and 10 age-matched able-bodied controls during quiet upright stance with eyes open, eyes closed and during dual tasking. To analyse afferent and efferent corticomuscular coherence, directional wavelet coherence between EEG and EMG signals was computed. The corticomuscular coherence analysis showed significant differences between amputees and controls in the afferent and efferent direction and across visual conditions, suggesting differences in the processing of sensory feedback. A power spectral density analysis of the motor cortex contralateral to the amputated leg of amputees showed increased power, as well as a pronounced decrease in alpha frequencies indicating an increased cognitive load. This exploratory study stimulates further hypotheses on how coordination of brain and muscle activity is modulated after a lower limb amputation.

## Teaser

Patients with a lower limb amputation adapt their neuromuscular system to maintain balance. Meyer *et al*. noted changes in EEG power spectral density and directional corticomuscular coherence between the brain cortex and muscles of the lower body during quiet standing with and without vision and during dual tasking.

## Graphical Abstract

**Graphical Abstract:**
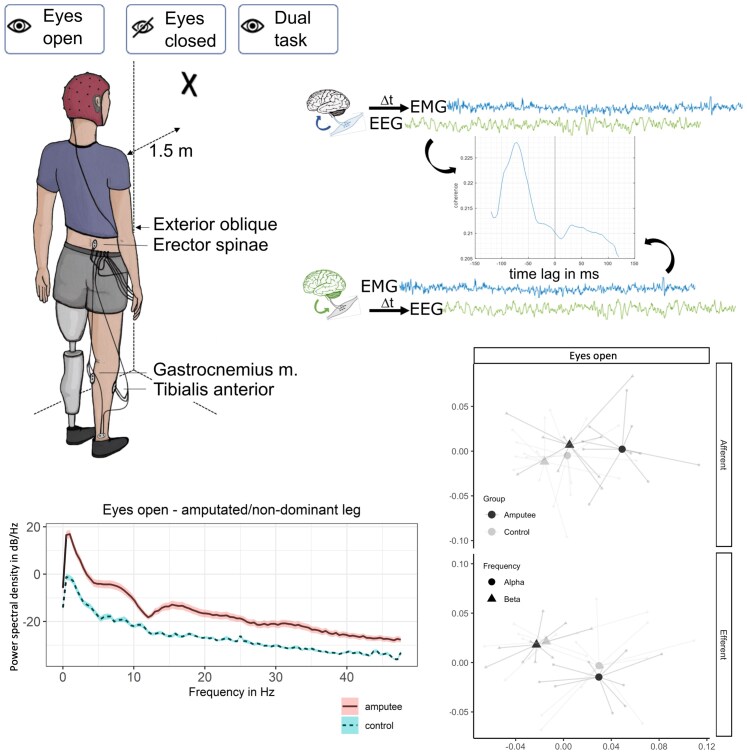

## Introduction

As human bipedal stance is an inherently unstable process, brain and muscles work continuously in concert to counterbalance gravity and larger perturbations.^1^ While these processes are natural and often unconscious for able-bodied individuals, for patients with lower limb amputations (especially above the knee) even unperturbed upright standing can be challenging. This is mainly due to neuromuscular adaptations occurring after an amputation as a result of disrupted sensory feedback loops and biomechanical alterations.^2,3^

The loss of sensory feedback from the amputated leg modifies the feedback control schema, resulting in an overuse of the intact limb and increased reliance on visual and vestibular feedback to control balance.^4^ For example, reduced intermuscular coherence^2^ and increased dynamics of the center of pressure oscillation in the intact leg of amputees^3,5^ have been observed during quiet standing when visual feedback is removed. Moreover, limb loss results in a reduced base of support and an asymmetric weight distribution,^4^ possibly as a consequence of lack of trust in the prosthetic limb. Thus, the intact leg compensates for the reduced flexibility and mobility of the prosthesis.

Biomechanically, this instability is mainly compensated by an increased use of a hip strategy,^1^ where the upper body counterbalances large perturbations. This stands in contrast to an ankle strategy, more often used by able-bodied individuals, during which small perturbations are compensated by activation of the muscles around the ankle joints.^6^ From a neurological perspective, a remapping in the cerebral cortex takes place over time as the original neural pathway is no longer functional. Changes in the motor and the somatosensory cortex after a lower limb amputation include reduced inter-hemispheric connectivity and increased connectivity between areas contralateral to the amputation.^7^ Thus, identifying possible alterations in the signal pathways between the cerebral cortex and muscles controlling the intact limb becomes relevant to understand the disrupted postural control of patients with a lower limb amputation.

One possible approach to this problem is to measure corticomuscular coupling^8^ by quantifying the neural synchrony between the motor cortex and muscles. Corticomuscular coherence (CMC) describes this synchrony by identifying frequency bands occurring simultaneously in electrical signals recorded from the brain [electroencephalogram (EEG)] and muscles [electromyogram (EMG)], reflecting afferent and efferent signalling pathways. CMC has been studied during different balance and force tasks in able-bodied individuals, linking the observed frequency band activity to physiological processes. For instance, CMC in the alpha band frequencies (8–12 Hz) has been associated with afferent or feedback pathways, as well as with error correction during tasks where participants have to rapidly match a given target force.^9^ CMC in the beta band (13–30 Hz) was observed while exerting sustained, isometric force^9,10^ and during quiet upright stance.^11^ CMC in the gamma band (low: 30–45 Hz; high: 45–63 Hz) has been frequently observed during dynamic tasks^9,10,12^ and during postural control.^11,13^ The gamma band is further associated with complex and challenging motor conditions.^12^

**Figure 1:**
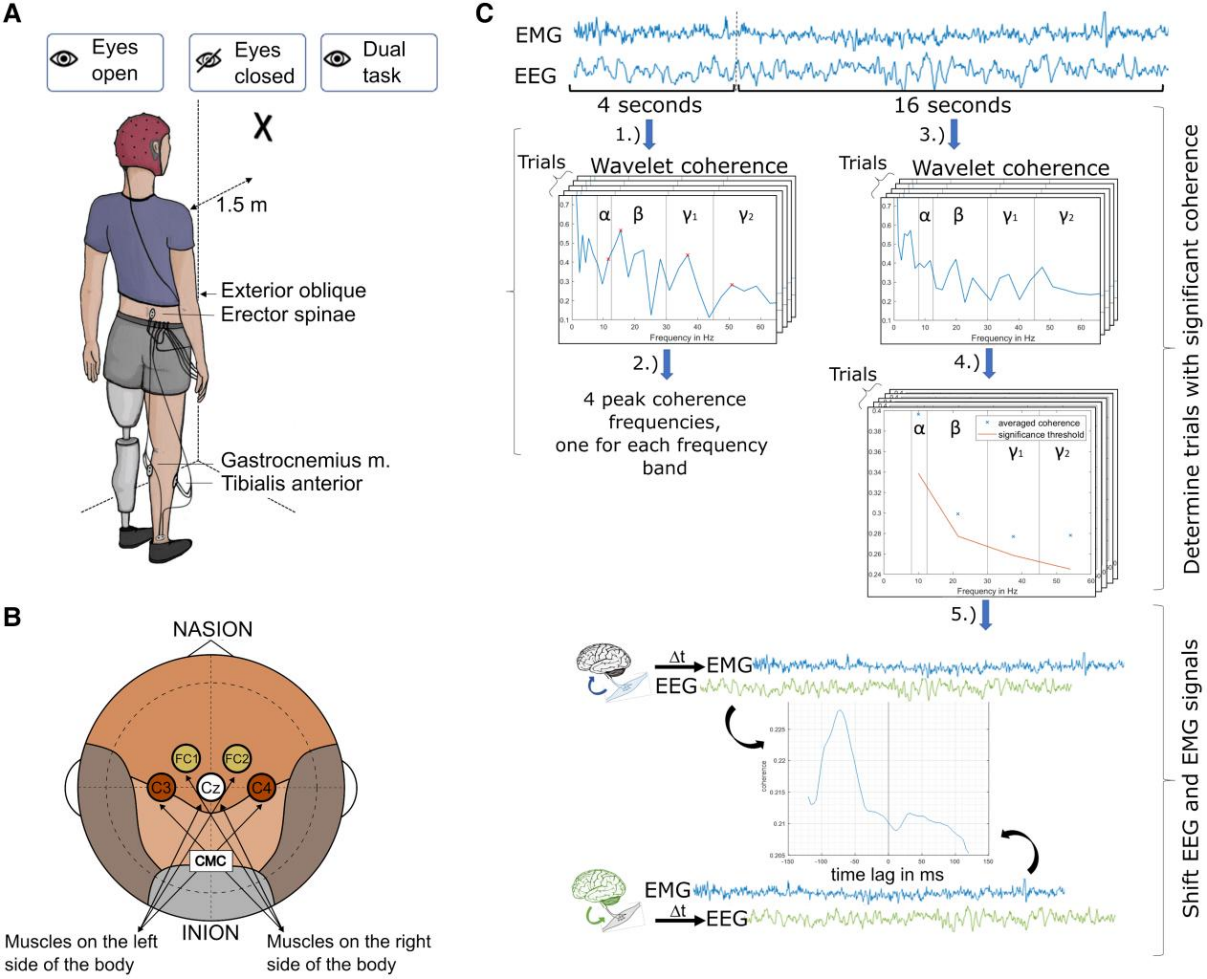
**Experiment and data processing description**. (**A**) Experimental setup. EEG and EMG signals were recorded during a quiet upright stance with three different tasks. (**B**) EEG electrodes used for the computation of CMC. The electrodes were positioned according to the 10–20 system, covering the motor cortex. (**C**) Processing pipeline for the CMC analysis. (1) The wavelet coherence from the first four seconds of every trial was computed and (2) used to determine peak coherence frequencies for all frequency bands of each participant. (3) From the residual, 16-s wavelet coherence for the peak frequencies was computed and (4) checked for significance. (5) To determine afferent and efferent coherence, the wavelet coherence was recomputed for time shifted EEG and EMG signals.

To the best of our knowledge, CMC in unilateral lower limb amputees during balance control has not yet been investigated. This study aims to fill this research gap by exploring for the first time CMC between the motor cortex and muscles driving the intact leg and the trunk of unilateral transfemoral amputees during quiet standing. Experimental conditions included visual feedback [eyes open (EO)], no visual feedback [eyes closed (EC)] and increased cognitive load by a dual task (DT) paradigm in which the subjects had to quietly count in steps of three (Fig. 1). Hemispherical differences in the power spectrum over the motor cortex between amputee patients and able-bodied individuals were further analysed. Due to the described alterations in the cortex and the control of muscles, we hypothesize that CMC and the power spectrum of amputees will differ from controls. Especially when removing visual feedback strong differences in CMC are expected, influencing alpha band coherence. Further, the usage of a prosthetic leg requires more attention to maintain balance. We hypothesize that the additional cognitive load of the duals task paradigm will lead to altered CMC in the gamma band of amputees.

## Materials and methods

### Participants

Ten unilateral transfemoral amputees participated in this study (age: 58.6 ± 11.8 years, height: 1.71 ± 0.13 m, weight with prosthesis: 77.1 ± 9.0 kg; four females and six males), who were recruited in collaboration with Pfänder Orthopedics, Freiburg, Germany. The detailed characteristics of participants with an amputation are shown in Table 1. Only patients without any other orthopaedic, neurological or cardiovascular conditions were included in the study. A control group of 10 age-matched able-bodied persons (age: 52 ± 14.8 years, height: 1.72 ± 0.08 m, weight: 75.1 ± 11.7 kg; six females and four males) also participated in this study. All participants had normal or corrected-to-normal vision. All participants signed an informed consent form, approved by the ethics committee of the University of Freiburg, Freiburg, Germany (ethical approval no. 230/18).

**Table 1:** Characteristics of amputated participants

ID	Age (years)	Height (cm)	Weight (kg)	Years since amputation	Prosthesis
1	68	180	81	32	OG
2	57	179	85	49	OG
3	65	187	70	37	OG
4	58	183	70	34	OG X3
5	63	158	76	43	O C-Leg
6	30	165	67	25	OG X3
7	59	170	92	40	OG
8	52	156	65	36	O C-Leg
9	60	164	80	4	O C-Leg
10	73	165	85	5	O Kenevo

O, Ottobock; OG, Ottobock Genium.

### Experimental design and data collection

Participants performed seven 30-s trials of quiet upright standing for each of three tasks (Fig. 1A), including EO, EC and a DT. During the EO task, participants fixated a cross, positioned at 1.5 m distance at eye-level. The DT condition was also conducted with EO and included backward quiet counting in steps of three, starting with a positive number that varied around 150. The trial order was chosen such that participants performed always all of the three task types in a row, until each task was performed seven times. The order of each block of the three tasks was randomized using a computer-generated sequence. A short break of approximately 1 min was administered after seven consecutive trials, in which participants rested on a chair. During each trial, participants stood with their feet shoulder-to-shoulder width apart, and with their arms hanging relaxed close to their body.

Bipolar surface EMG signals were recorded from four muscles known to be involved during the control of upright standing.^14-17^ Disposable, adhesive, gel-based silver/silver chloride electrodes were used. These included the M. obliquus externus (ExO), M. erector spinae (ES), M. tibialis anterior (TA) and M. gastrocnemius medialis (GM). Reference electrodes were attached to the malleolus bones at the ankle. All EMG electrodes were attached to the dominant leg of controls or the intact leg of amputees. The dominant leg of controls was defined by asking with which foot they step first during walking or climbing stairs. The recording was performed with the BIOPAC MP35 system (BIOPAC, CA, USA) with a sampling frequency of 2000 Hz. Electrodes were placed according to the SENIAM project guidelines after cleaning the skin with alcohol to remove dead skin particles and oil residues.^18^

EEG signals were recorded simultaneously using a 32-channel dry electrode cap waveguard™touch (ANT Neuro, Hengelo Netherlands). The cap was fitted according to the international 10–20 standard with ground and reference electrodes attached to the mastoids. Impedance measurements and recordings were performed with the eego™ software from ANT Neuro. The recordings were performed with a sampling frequency of 500 Hz and synchronized with the EMG recordings using an external common trigger.

### Data processing

The general workflow of this study is described as follows (detailed information follows in the next subsections). After pre-processing (filtering and down sampling), CMC was computed between EEG and EMG signals using the wavelet coherence. For the correct computation of CMC, we further estimated the delay between detecting cortical and muscle activation and shifted the signals accordingly.^19,20^ Depending on the direction of the shift, directional CMC was computed for afferent (i.e. muscle to cortex) and efferent (i.e. cortex to muscle) directions. A significant threshold was further computed using surrogate analysis to analyse only the trial where the CMC values are statistically higher than random processes.

#### Signal pre-processing

Data processing was performed using Matlab version 2019b (MathWorks, Natick, MA, USA) and the MATLAB-based toolbox EEGLAB.^21^ A Butterworth notch filter of second order was applied to remove 50-Hz noise of EEG and EMG data. Further, both datasets were high-pass filtered with a cut-off frequency of 1 Hz and low-pass filtered with a cut-off frequency of 100 Hz. The first and last 5 s of each measurement were neglected to avoid any transients. Additionally, the EMG signals were cleaned of ECG contaminations by computing the intensities of the EMG signals in wavelet space^22^ and subtracting a thereby reconstructed and smoothed ECG signal from the EMG data. EEG data were re-referenced with a common average reference. Both EMG and EEG signals were downsampled to 250 Hz. The largest artefacts within the EEG data were removed using wavelet transforms as described before, residual artefacts were removed through independent component analysis using the Infomax algorithm implemented in EEGLAB.^21^

Data analysis was performed for five electrodes, covering as much as possible the motor cortex (Fig. 1), including the Cz electrode and electrodes contralateral to the dominant or intact leg as FC1/FC2 (termed as frontal electrodes in this work) and C3/C4 (termed as central electrodes in this work). As postural control is a more demanding and dynamic task for amputees, the frontal electrodes were included to capture possible differences during movement planning. The central electrodes should cover representations of trunk muscles; however, a recording of somatosensory signals cannot be excluded or prevented due to the spatial resolution of our EEG system.

#### Corticomuscular coherence

CMC between windowed EMG and EEG data was calculated using a wavelet-based coherence analysis. The procedure to calculate the wavelet coherence is explained in detail in a previous publication.^2^ Briefly, first the wavelet transform of the EMG and EEG signals is obtained, yielding a time–frequency representation of the signals at a distinct center frequency (cf). The wavelet transform is computed by the convolution of each signal with a set of non-linearly scaled wavelets. The mother wavelet used to for this set was the complex Cauchy, with the different wavelets in the frequency space defined by Eq. (1) and Eq. (2).


(1) $$F\psi (\;f,cf,\mathrm{mode})={\left(\frac{\;f}{cf}\right)}^{\mathrm{mode}}\;\cdot \;e^{\left(\frac{-f}{cf}+1\right)\cdot \mathrm{mode}}$$

(2) $$cf_{j}=\frac{1}{\mathrm{scale}}\cdot (\;j+q{)}^{r}$$

In Eq. (1), *f* is the frequency in the Fourier frequency space and mode is defined as cf⋅scale. Hence, the wavelets are functions of the scaling factor that influences the frequency range and the cfs, which indicate the maximum of the wavelet in frequency space.^22^ The cfs were defined by Eq. (2), where a scale of 12 was used, *j* ranged from 1 to 30 and the parameters *q* and *r* were set to 1.45 and 1.959, respectively.^22^ This resulted in 30 cfs from 0.5 to 67 Hz. The wavelet coherence was then calculated as the modulus of the wavelet coherency (Eq. 3), averaged over all windows.


(3) $$\mathrm{coherency}=\frac{W_{x}\;*\;{\bar{W}}_{y}}{{\left(W_{x}\;*\;{\bar{W}}_{x}\right)}^{0.5}*\;{\left(W_{y}\;*\;{\bar{W}}_{y}\right)}^{0.5}}$$

In Eq. (3), $W_{x}$ and $W_{y}$ are the wavelet transforms and their conjugated complex counterparts of the signals *x* and *y.*^23^ A window length of 2 s without overlap was chosen. Due to an overall signal length of 15.87 s, this resulted in a frequency resolution of 0.504 Hz. Each window was zero-padded at both extremes. Within each window, the wavelet coherence was averaged for each cf over time.

#### Estimation of transmission delay and direction between brain and muscles signals

For the correct computation of coherence between EEG and EMG signals, the transmission delay between signals from the brain and muscles was removed. This was performed by adapting the method from Xu *et al*., whereby EEG and EMG signals are shifting against each other until the coherence reaches its maximum.^19,20^ Hence, the time shift needed to reach the maximum represented thereby the time delay. The estimated time delays ranged for all muscles in afferent transmission direction between 24.12 ± 13.31 ms and 26.80 ± 13.09 ms, while for the efferent direction from 21.91 ± 12.00 ms to 23.7 ± 12.10 ms (Table 2). Our estimated delays agree with latencies of motor evoked potentials and theoretical calculations in the literature.^24-26^ Additionally, this method allows to distinguish afferent and efferent signals, depending on how the datasets are shifted against each other (afferent: EMG signals antecede EEG signals; efferent: EEG signals antecede EMG signals, Fig. 1B, Step 5). However, to obtain a precise estimation of the time delay, signals of only one transmission pathway (i.e. afferent or efferent) would be needed instead of the recorded mixture of multiple pathways. To minimize the distortion of the time delay calculation by these mixed signals, in a first step the peak frequencies with significant coherence were determined for every participant. Thus, the first four seconds of every trial were used to calculate the average peak coherence frequencies within the alpha (8–12 Hz), beta (13–30 Hz), low gamma (30–45 Hz) and high gamma (45–63 Hz) bands (Fig. 1B, Steps 1 and 2). This allowed us to independently search in the next 16 s for these particular frequencies as a representative of each frequency band. We used the first 4 s to avoid bias in the finding of the peak frequencies. Subsequently, the remaining 16 s of EEG and EMG signals were used to determine trials and frequency bands with significant coherence. For that, the wavelet coherence of every trial was computed (Fig. 1B, Step 3) and the averaged coherence of each frequency band was compared with a significance threshold, calculated through a surrogate analysis explained in the next subsection (Fig. 1B, Step 4). Only trials and frequency bands above this significance threshold were used for the final calculation of afferent and efferent CMC. Finally, the EEG and EMG signals were shifted against each other (Fig. 1B, Step 5) and the coherence for the predetermined peak frequencies (calculated in Fig. 1B, Step 2) was recomputed. Through this, the point of maximal coherence could be calculated, providing the time delay and the direction of signal transmission. The signals were shifted until a 128 ms time difference in afferent and efferent direction was reached. The signal length was kept constant at 15.872 s (16–0.128 s). Through an additional moving average over 16 ms, the resulting curve was smoothened, obtaining a clearer peak for the time delay calculation.

**Table 2:** Averaged time delay between EEG signals (central, cz and frontal) and EMG signals from trunk (ExO and ES) and leg muscles (TA and GM)

		Trunk		Leg	
		Amputee	Control	Amputee	Control
Afferent	Frontal	24.59 ± 12.92	24.18 ± 13.20	25.05 ± 13.31	24.12 ± 13.31
	Cz	24.85 ± 12.62	26.80 ± 13.09	24.69 ± 13.69	26.25 ± 12.76
	Central	26.09 ± 13.24	24.34 ± 13.30	24.92 ± 13.09	24.65 ± 13.27
Efferent	Frontal	22.22 ± 12.36	22.58 ± 12.56	21.91 ± 12.00	22.79 ± 12.60
	Cz	22.47 ± 12.22	23.19 ± 12.40	22.43 ± 12.79	22.84 ± 11.82
	Central	22.14 ± 12.34	18.17 ± 14.58	23.44 ± 12.87	23.7 ± 12.10

The table shows mean and standard deviation of the latencies in milliseconds.

#### Power spectral density

We estimated the power spectral density (PSD) of the EEG signals from the frontal, central and the Cz electrodes for amputees and able-bodied controls. PSD was calculated from zero-padded boxcar windows of 2 s length.

### Statistical analysis

#### Significance threshold

Significance thresholds were calculated using surrogate analyses to determine if the coherence values of each trial were statistically significantly different than coherence values obtained by chance. Thus, surrogates of the pre-processed EEG and EMG datasets were calculated by using the amplitude-adjusted Fourier transform (AAFT) method^27^ (Fig. 1B, Step 4). The AAFT technique alters the signal by randomizing its phase, effectively eliminating its informational content while preserving its amplitude properties. This process generates a new signal that resembles the original but lacks any meaningful information. When applied to EMG or EEG data, it mimics the effect of collecting these signals in a completely random manner. Consequently, coherence values that are statistically higher than those derived from EMG and EEG data after the AAFT process can be regarded as significantly exceeding chance levels and, therefore, as meaningful. To increase the robustness of this method, 100 surrogates were computed, from which the median surrogate was calculated and used to compute the CMC significant threshold.

#### Multivariate analysis

The data were averaged across the seven trials (resulting in one CMC value per participant, task and frequency band) and split in two groups, one consisting of CMC values from the pairs comprising all the EEG electrodes (Central, Cz and Frontal) and EMG electrodes from the trunk muscles (ExO and ES), and the other comprising the CMC values from the pairs of all the EEG electrodes (Central, Cz and Frontal) and the EMG electrodes from the leg muscles (TA and GM). Thus, each group (trunk and leg) contains vectors of CMC values, each of six dimensions (3 EEG electrodes × 2 EMG electrodes). We repeated this process for each direction (afferent and efferent direction). This allowed the analysis of CMC between the brain cortex and trunk and leg independently for each direction.

Permutational multivariate analysis of variance (PERMANOVA)^28^ was then applied on CMC profiles of the two groups (trunk and leg) separately to investigate the main fixed effects of group (two levels: amputees and controls), task (three levels: EO, EC and DT) and frequency band (two levels: alpha and beta). The random effect was set to the individual participant groups. We used altGower as distance metric with 5000 permutations. The statistical threshold for significance was set to *α* = 0.05. Nonmetric dimensional scaling (NMDS) was used to visualize the six-dimensional CMC profiles in two dimensions.^28^ All statistical analysis was performed using R.^29^

## Results

### Power spectrum density analysis

**Figure 2:**
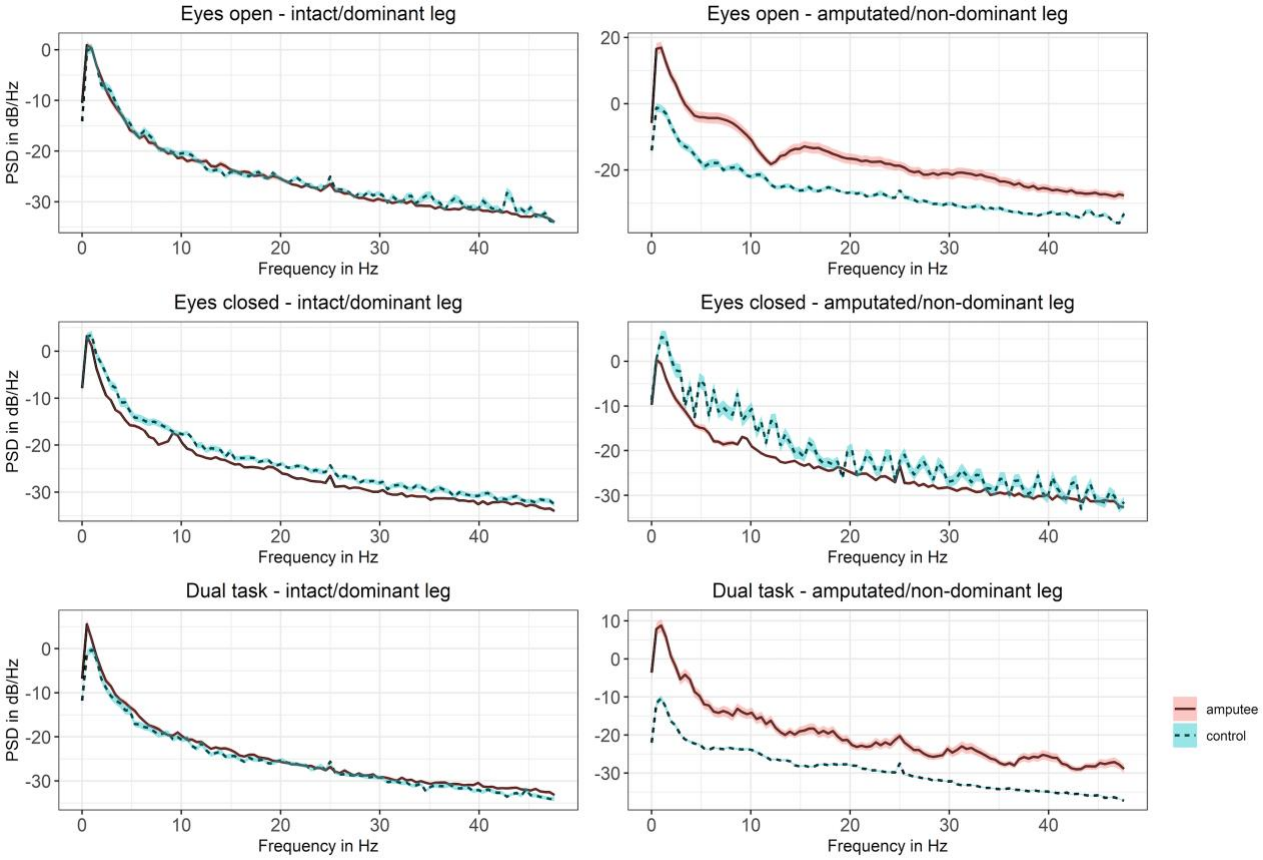
**PSD of frontal EEG electrodes.** Each graph shows the PSD (black line) and the standard error of the mean (shaded area) for amputees in pink and controls in blue. The left column shows signals of the brain hemisphere representing the intact leg of amputees and the dominant leg of controls, whereas the hemisphere controlling the amputated leg of amputees and the non-dominant leg of controls is shown in the right column. From top to bottom trials with EO, EC and the DT are shown. Control group, *n* = 10, amputee group, *n* = 10.

First, we examined the power spectrum density (PSD) from frontal channels of both hemispheres of amputees and controls (Fig. 2). The largest difference between amputees and controls was observed in the electrode signals contralateral to the amputation or the non-dominant leg of controls. During the EO tasks (visual feedback available), the PSD of amputees showed a pronounced decrease in the alpha band, as well as an overall higher power. The PSD analysis also revealed that DT (additional cognitive load) elicited higher power for amputees compared with controls. Increased activity of amputees in comparison with controls was also observed for the central channels during all tasks on the contralateral side to the amputation (Supplementary Fig. 1). No distinguishable differences between groups were observed on the Cz EEG channel (Supplementary Fig. 2). These findings show that unilateral lower limb amputations lead to significant structural changes in the motor cortex contralateral to the amputated leg.

### CMC analysis

CMC was computed between frontal (FC1/FC2), Cz and central (C3/C4) EEG channels and EMG signals of the external oblique (ExO), the ES, the shin TA and the medial gastrocnemius (GM) muscles of the intact leg of amputees or dominant leg of controls (Supplementary Fig. 3). A large amount of the CMC values corresponding to low and high gamma frequency bands were below the significance threshold (low gamma: 41.73%; high gamma: 82.15%), thus these two frequency bands were not analysed further. For the alpha and beta frequency bands, the percentage of non-significant CMC values was 7.29% and 14.58%, respectively. Peak CMC values within the alpha and beta band are listed in Supplementary Table 1.

The CMC values from the EEG channels (Central, Cz and Frontal) and the EMG channels from the trunk muscles (ExO and ES) were grouped to form a six-dimensional vector. This procedure was repeated for the CMC values from the same EEG channels and the EMG channels from the leg muscles (TA and GM). We performed multivariate analysis on these vectors, which allowed the analysis of CMC profiles between brain cortex and trunk/leg muscles independently.

#### CMC between EEG signals and EMG signals from the trunk

**Figure 3:**
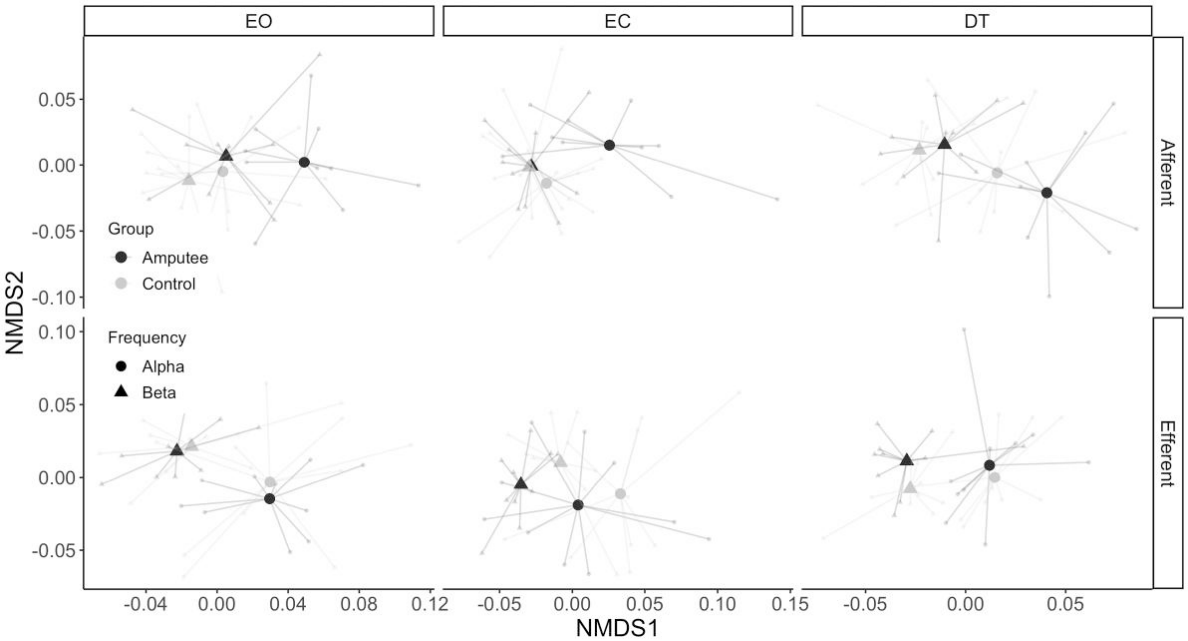
**Nonmetric dimension scaling analysis (NMDS) for the CMC values between EEG signals (central, cz and frontal) EMG signals from trunk (ExO and ES) muscles**. In this plot, each point represents the six-dimensional CMC profile of each participant (light small dots). Centroids for each group are represented with larger symbols (large triangle and dots). Points that are closer to each other present more similar CMC profiles, while points that are away from each other present more different CMC profiles. PERMANOVA was applied on CMC profiles with main fixed effects of group (amputees and controls), task (EO, EC and DT) and frequency band (alpha and beta). The random effect was set to the individual participant groups. *P*-values are reported in Tables 3 and 4. Control group, *n* = 10, amputee group, n = 10. EO, eyes open; EC, eyes closed; DT, dual task.

The CMC profiles for the trunk muscles were analysed in the afferent and efferent direction (Fig. 3). A significant effect of frequency band was observed for both directions (*P*-value < 0.001, Table 3). In general, alpha band CMC was higher than the beta band CMC (Supplementary Fig. 3). A significant effect of group was observed for the afferent direction (*P*-value < 0.001, Table 3), but there were no significant effects of task nor interactions (Table 3). Supplementary Fig. 3 shows that the amputee group elicited higher afferent CMC for most of the electrode pairs. A significant effect of group (*P*-value < 0.001, Table 3) and a significant group x task interaction effect were observed for the efferent direction (*P*-value = 0.010, Table 3), where the CMC values in the EC task (no visual feedback) differed significantly between amputees and controls (*P*-value = 0.003, Table 4). The findings imply that the neural transmission of patients with an amputation maybe modulated by visual feedback.

**Table 3:** Multivariate analysis using PERMANOVA on CMC values between EEG signals (central, cz and frontal) and EMG signals from trunk (ExO and ES) and leg muscles (TA and GM)

	Trunk				Leg			
	Afferent		Efferent		Afferent		Efferent	
	F	*P*-value	F	*P*-value	F	*P*-value	F	*P*-value
Frequency	**9**.**67**	**<0**.**001**	**15**.**64**	**<0**.**001**	**14**.**02**	**<0**.**001**	**11**.**18**	**<0**.**001**
Task	1.02	0.397	0.88	0.543	0.70	0.697	0.91	0.514
Group	**4**.**73**	**0**.**001**	**2**.**39**	**<0**.**001**	**1**.**91**	**<0**.**001**	**0**.**98**	**<0**.**001**
Frequency:Task	0.95	0.464	1.41	0.187	0.74	0.653	0.93	0.498
Frequency:Group	0.70	0.615	0.69	0.619	0.26	0.901	0.29	0.898
Task:Group	0.65	0.746	**2**.**38**	**0**.**015**	1.17	0.294	**2**.**16**	**0**.**021**
Frequency:Task:Group	1.14	0.303	0.83	0.584	0.63	0.756	1.53	0.130

The CMC values from these six pairs of electrodes were grouped in single six-dimensional vector. PERMANOVA performs a multivariate analysis based on similarity metrics between CMC profile vectors. The table shows *F*- and *P*-values for factors affecting the similarity between CMC profile vectors. Statistical significances (*P*-value < 0.05) are indicated in bold.

**Table 4:** Multivariate analysis using PERMANOVA on the task:group interaction effect in the efferent direction for the CMC profiles between EEG signals (central, cz and frontal) and EMG signals from trunk (ExO and ES) and leg muscles (TA and GM)

	Trunk		Leg	
	F	*P*-value	F	*P*-value
EO	1.08	0.371	**3**.**69**	**0**.**002**
EC	**4**.**21**	**0**.**002**	0.05	0.973
DT	0.67	0.633	1.38	0.230

Results shows comparison between groups (amputees versus control) for the three tasks (EO, eyes open; EC, eyes closed; DT, dual task). Statistical significances (*P*-value < 0.05) are indicated in bold.

#### CMC between EEG signals and EMG signals from the leg

**Figure 4:**
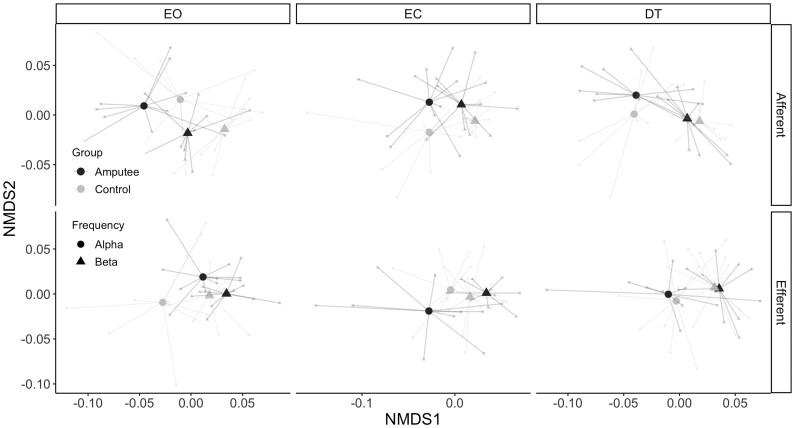
**Nonmetric dimension scaling analysis (NMDS) for the CMC values between EEG signals (central, cz and frontal) and EMG signals from leg (TA and GM) muscles**. In this plot, each point represents the six-dimensional CMC profile of each participant (light small dots). Centroids for each group are represented with larger symbols (large triangle and dots). Points that are closer to each other present more similar CMC profiles, while points that are away from each other present more different CMC profiles. PERMANOVA was applied on CMC profiles with main fixed effects of group (amputees and controls), task (EO, EC and dual task) and frequency band (alpha and beta). The random effect was set to the individual participant groups. *P*-values are reported in Tables 3 and 4. Control group, *n* = 10, amputee group, *n* = 10. EO, eyes open; EC, eyes closed; DT, dual task.

The CMC profiles for the leg muscles were also examined in the afferent and efferent direction (Fig. 4). As for the trunk muscles, a significant effect of frequency band was present for both directions (*P*-value < 0.001, Table 3), showing in general higher alpha band CMC compared with beta band CMC (Supplementary Fig. 3). For the afferent direction a significant effect of group was observed (*P*-value = 0.015, Table 3), but there were no significant effects of task nor interactions (Table 3). Supplementary Fig. 3 shows that the amputee group elicited higher afferent CMC for most of the electrode pairs. A significant effect of group (*P*-value < 0.001, Table 3) and a significant group × task interaction effect were present for the efferent direction (*P*-value = 0.043, Table 3), with the CMC values differing significantly between the group of amputees and controls in the EO task (*P*-value = 0.027, Table 4). These results suggest a reduced cortical control of muscles of the lower leg in patients with an amputation.

## Discussion

This study investigated alterations in corticomuscular coupling during balance control after a unilateral transfemoral amputation. The results from the PSD and CMC analyses evidenced pronounced differences between patients with an amputation and able-bodied individuals, modulated by visual feedback.

The overall signal power of the hemisphere contralateral to the amputated leg was considerably higher than for the non-dominant leg of the control group. Increased cortical activity contralateral to the amputation was reported in an upper limb study whilst moving the intact hand^30^ and compared with a control group.^31^ Simões and colleagues also observed an increased activity contralateral to the amputation compared with controls after sensory stimulation of the foot and the stump of lower limb amputees.^32^ This unexpected activity is often explained by a reduced inter-hemispheric inhibition, resulting from the deafferentation and increased use of the intact limb.^30,31^ Additionally, Makin and colleagues reported that the reliance on the intact limb increases its cortical representation within the ipsilateral cortex of amputees.^33^

The PSD of the frontal channels over the cortex area representing the amputated leg exhibited a strong decrease in the alpha band. This decrease was not observed in the Cz and central channels and thereby might be associated with movement planning. The decrease in the alpha band was also only present for the EO task, suggesting an association with the processing of visual feedback rather than reflecting the lack of proprioceptive feedback. These findings are in line with studies reporting an alpha power decrease during tasks requiring visual attention.^34-36^ The lateralized alpha decrease is believed to reflect the active processing of a task, whereas task-irrelevant brain regions are functionally inhibited by increased alpha oscillations.^37-41^ Parr *et al*. tested changes in the alpha power during a grasp and lift task when naive subjects used a hand prothesis simulator compared with using their actual hand, and observed a stronger alpha decrease on central and parietal channels during the prothesis usage.^42^ They concluded that a decreased alpha power reflects an increased cognitive burden, which is associated with the required visual attention.^42^ Our findings might thereby reflect the increased need of visual information and a resulting cognitive load regarding the amputated leg.

Our results also suggest a different modulation of visual feedback over CMC of the intact leg in the amputee group compared with able-bodied controls (significant interaction effect of task and group in the efferent direction, Table 3 and Table 4). This was observed regardless of the frequency band for the CMC values in the efferent direction to the trunk muscles. During tasks with closed eyes, the amputee group showed mostly lower coherence between the cortex and trunk muscles compared with the control group. This implies that the efferent control of the trunk muscles of lower limb amputees is mainly influenced by visual information. Contrary to our hypothesis, no significant interaction effects with the frequency bands were found.

Our results did not show significant CMC in afferent direction, implying a unidirectional visual feedback modulation from the cortex to muscles. However, there was a significant difference in CMC between amputees and controls in both direction (Table 3), which indicates that afferent corticomuscular coupling, even though it seems not to be modulated by visual feedback, differs between amputees and controls. This may have consequences in how sensory feedback is processed in the amputee group.

Moreover, our CMC results also reflect the different usage of ankle and hip strategies. The significant interaction effect of the leg muscles suggests that during normal standing controls have an increased reliance on an ankle strategy to maintain balance. Only under more challenging conditions, when the eyes are closed, controls also seem to adopt a hip strategy, resulting in increased CMC of the trunk muscles (Supplementary Fig. 3).

There were significant differences between CMC values in the alpha and the beta frequency ranges regardless of group, direction or task (no interaction effects, Table 3), with CMC values being higher in the alpha band (Supplementary Fig. 3). Thus, these results suggest that the different modulation of visual feedback on corticomuscular coupling observed in amputees and able-bodied individuals during quiet standing are not frequency-specific. In general, alpha band coherence has been linked in the literature to feedback processing^9^ and the beta band has been associated with steady force outputs.^9,11^ Further, most of our CMC results were not significant in the low and high gamma frequency range. As EMG activity in the gamma band (around 40 Hz) has been associated with dynamic and challenging sensorimotor tasks, driven by corticospinal input,^12,43^ our experimental design of quiet standing may not have been sufficiently challenging to detect cortical to muscular drive in this frequency band. Against our expectations, the additional cognitive load of the DT in patients with an amputation also did not induce gamma band coherence. Fili *et al*. observed similar results in able-bodied participants.^12^ A cognitive DT paradigm did not elicit intermuscular gamma band coherence, whereas increased motor challenges during walking led to significant results.^12^

The experiments in our study were conducted during quiet upright standing, adding only slight difficulty to the motor task by including an EC and a counting condition. Future studies should increase the complexity of the motor tasks, by including a perturbation condition during standing or even walking to confirm and enhance the differences we reported here between the amputee and control groups. Previous studies successfully implemented more dynamic tasks, such as targeted walking and walking with partial visual restriction^12^ or rapid changes of a force output.^9^ In addition, manipulation of attention level via inclusion of augmented visual feedback might provide further insights into altered control strategies and cognitive load during standing and walking after lower limb amputation. Importantly, age might be a relevant factor in this exploratory study.^44^ Young participants might elicit different results than the adult and senior adult individuals participating in this study. For example, Bayram and colleagues found significantly decreased CMC in older participants during voluntary motor performance,^45^ and Johnson and Shinora observed different frequency band activity between young and old participants.^46^ Additionally, the time since the amputation might influence CMC, as balance control in general also improves with the time since amputation.^3^ In this study however, all except two participants had been amputated for at least 25 years. Alterations in the CMC due to the time since amputation might therefore be neglectable.

Results from this study are relevant for furthering our understanding on how the loss of a limb alters neuronal circuits at the central and peripheral levels of the nervous system and results in adaptations in control strategies. Our findings emphasize the importance of developing prosthetic devices with a sensory feedback system. This would reduce the reliance on visual feedback and the resulting cognitive burden in patients with an amputation. Furthermore, confidence in the prosthesis will improve, which could help to restore balance control similar to the one of able-bodied individuals. Methods and results might also be valuable to better understand changes in gait and posture after hemiparesis due to stroke and might be useful for adapting associated rehabilitation paradigms.

## Supplementary Material

fcaf238_Supplementary_Data

## Data Availability

The datasets generated during and/or analysed during the current study are available from the corresponding author on reasonable request. The MATLAB and R codes used in this study are available through the repository 10.5281/zenodo.15534028.
