# Supplementary material for: Corticomuscular coherence during upright standing in unilateral transfemoral amputees
Source: Brain Commun. 2025 Jun 14;7(3):fcaf238. doi: 10.1093/braincomms/fcaf238 (PMC12198670; doi:10.1093/braincomms/fcaf238)
Supplement: fcaf238_Supplementary_Data [file fcaf238_supplementary_data.pdf]

Supplementary Materials

Corticomuscular coherence during upright standing in unilateral transfemoral amputees

B. Meyer[1,2], T. Krauskopf[1,3], K. Fuchs[1], M. Beusterien[1], L. Klein[4], M. Mueller[5], T. Ball[3,6], G.W. Herget[4], N. Mrachacz-Kersting[2,3], V. von Tscharner[7], C. Mehring[3,8,9], Stieglitz[1,3,9†], C. Pasluosta[1,3†]

Supplementary Table 1: Averaged peak coherence frequencies for the alpha and beta band. The peak coherence frequencies were calculated using a wavelet-based coherence analysis. The alpha band contained three different center frequencies between 13.39 Hz and 11.91 Hz, the beta band contained eight center frequencies from 13.39 Hz to 30.75 Hz.

			Trunk		Leg	
			**Amputee**	**Control**	**Amputee**	**Control**
**Alpha**	**Afferent**	Frontal	9.74 ± 0.78	9.59 ± 0.37	9.67 ± 0.7	9.59 ± 0.38
		Cz	9.43 ± 0.57	9.56 ± 0.64	9.44 ± 0.56	9.56 ± 0.64
		Central	9.54 ± 0.44	9.74 ± 0.79	9.61 ± 0.58	9.69 ± 0.84
	**Efferent**	Frontal	9.69 ± 0.82	9.59 ± 0.37	9.63 ± 0.74	9.59 ± 0.37
		Cz	9.56 ± 0.64	9.56 ± 0.65	9.54 ± 0.44	9.55 ± 0.67
		Central	9.5 ± 0.5	9.58 ± 0.78	9.5 ± 0.5	9.44 ± 1.01
**Beta**	**Afferent**	Frontal	19.26 ± 2.2	19.18 ± 1.79	19.31 ± 2.23	18.82 ± 1.92
		Cz	18.33 ± 2.02	19.01 ± 1.9	18.27 ± 2.05	19.1 ± 1.79
		Central	18.85 ± 2.28	18.49 ± 1.98	18.65 ± 1.92	18.81 ± 2.06
	**Efferent**	Frontal	19.35 ± 2	18.96 ± 1.67	18.62 ± 1.73	19.01 ± 1.78
		Cz	17.93 ± 1.86	18.98 ± 1.76	18.96 ± 1.91	18.81 ± 1.85
		Central	18.85 ± 2.19	18.73 ± 1.84	18.62 ± 2.17	18.85 ± 1.99

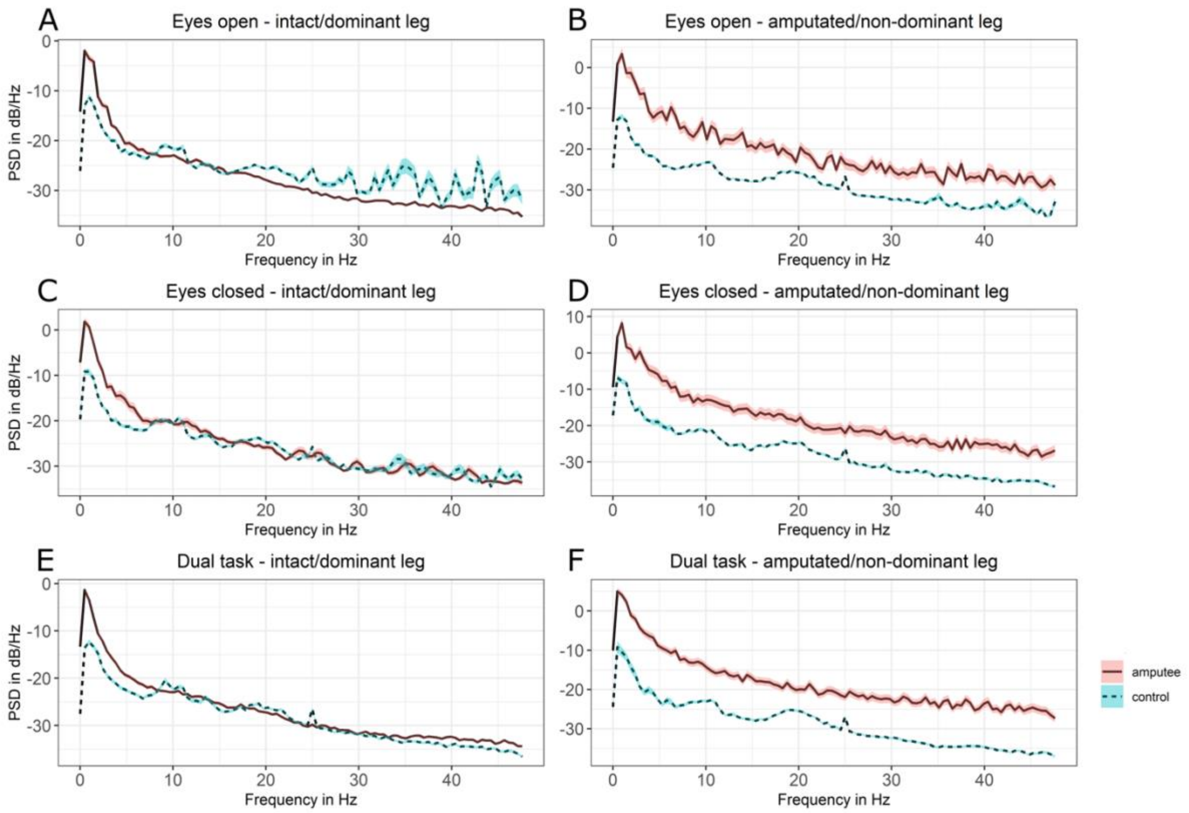

Supplementary Figure 1: PSD of central EEG electrodes. Each graph shows the PSD (black line) and the standard error of the mean (shaded area) for amputees in pink and controls in blue. The left column shows signals of the brain hemisphere representing the intact leg of amputees and the dominant leg of controls (A, C and E). The right column shows the hemisphere controlling the amputated leg of amputees and the non-dominant leg of controls (B, C and E).. From top to bottom: Trials with eyes open, eyes closed and the dual task. Control group, n = 10, amputee group, n = 10.

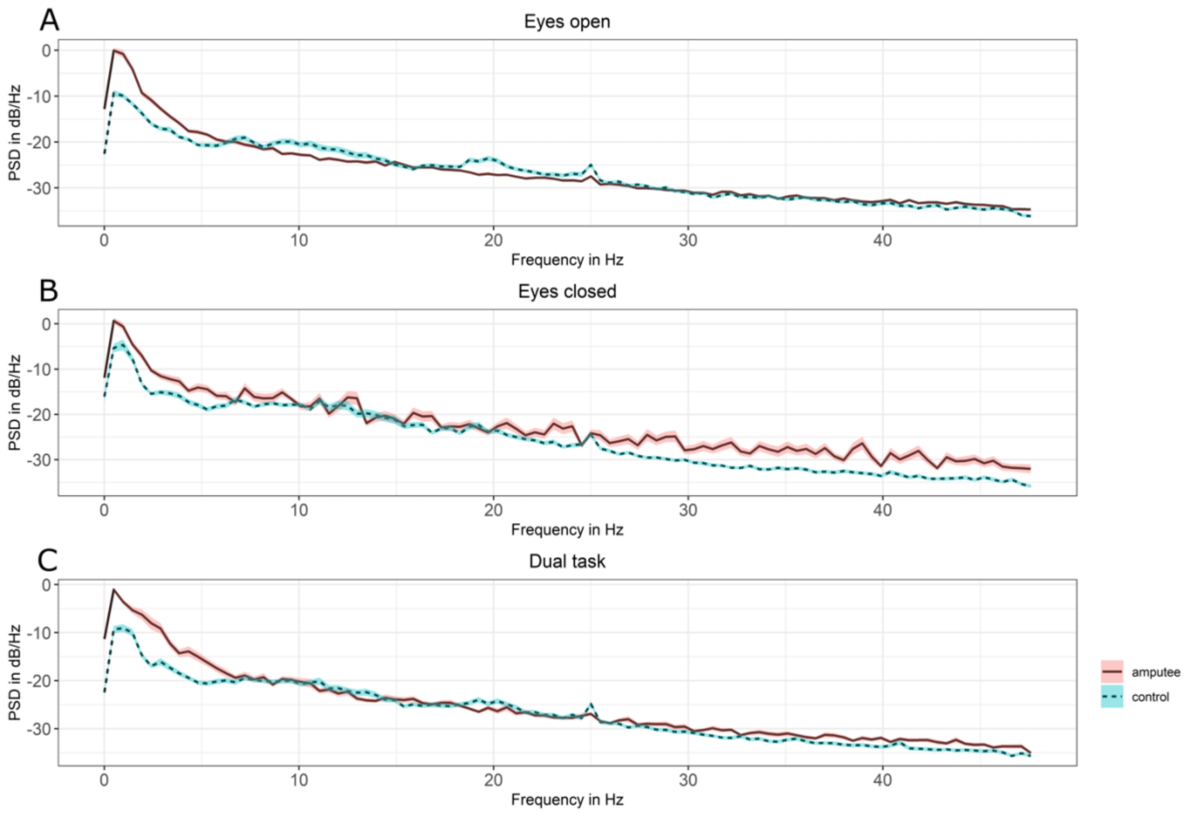

Supplementary Figure 2: PSD of Cz EEG electrode. Each graph shows the PSD (black line) and the standard error of the mean (shaded area) for amputees in pink and controls in blue. From top to bottom trials with eyes open, eyes closed and the dual task are shown. Control group, n = 10, amputee group, n = 10.

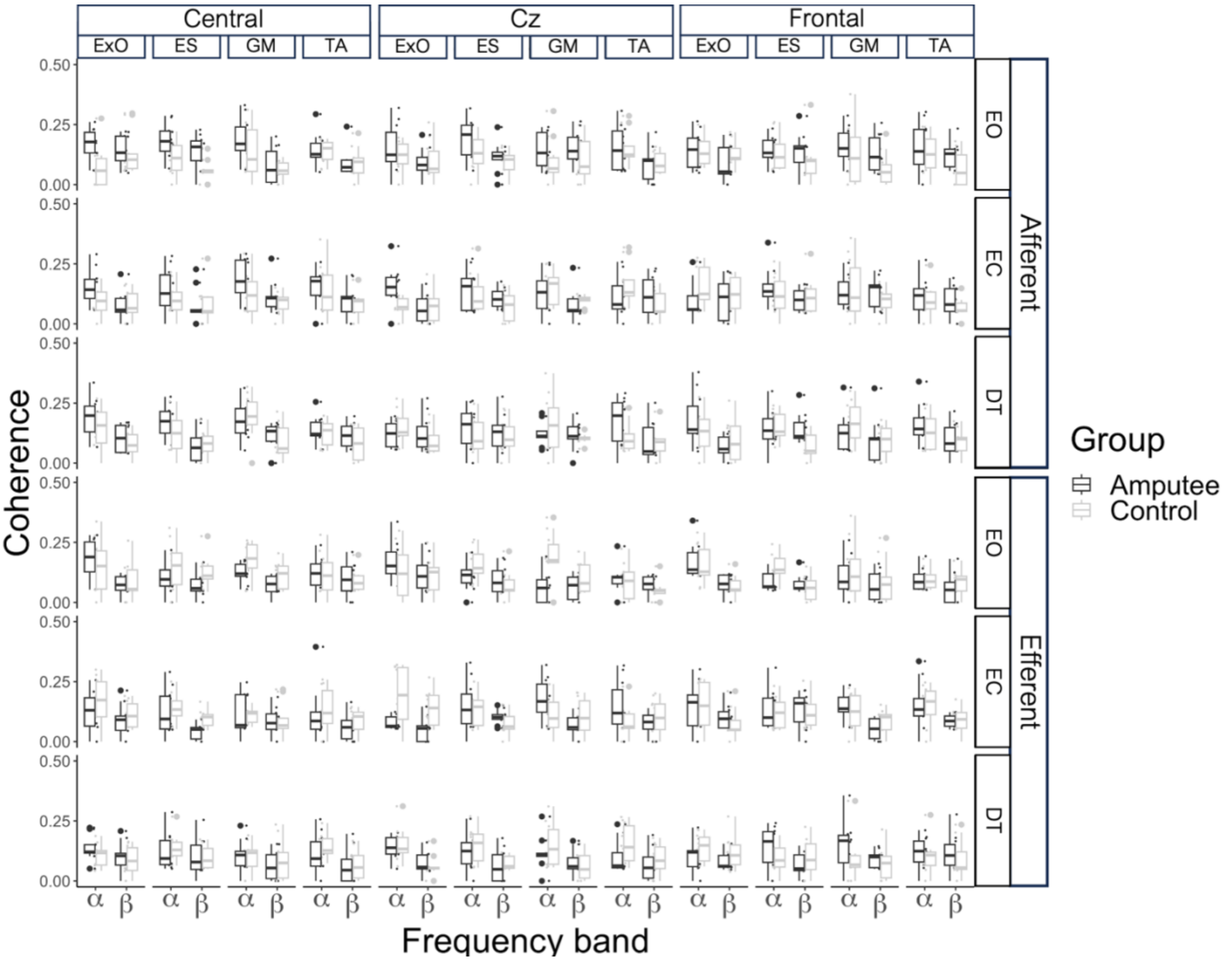

Supplementary Figure 3: Corticomuscular coherence between EEG channels (Frontal, Cz and Central) and EMG channels (ExO, ES, TA, GM). Summary of EEG-EMG coherence for afferent and efferent directions for the alpha (α) and beta (β) frequency bands in able-bodied control individuals (gray) and amputees (black).
